# Responsibility Gap(s) Due to the Introduction of AI in Healthcare: An Ubuntu-Inspired Approach

**DOI:** 10.1007/s11948-024-00501-4

**Published:** 2024-08-01

**Authors:** Brandon Ferlito, Seppe Segers, Michiel De Proost, Heidi Mertes

**Affiliations:** Department of Philosophy and Moral Sciences, Bioethics Institute Ghent, Ghent University, Blandijnberg 2, 9000 Ghent, Belgium

**Keywords:** Responsibility gap, Artificial intelligence, Ubuntu, Healthcare, Collective forward-looking responsibility

## Abstract

Due to its enormous potential, artificial intelligence (AI) can transform healthcare on a seemingly infinite scale. However, as we continue to explore the immense potential of AI, it is vital to consider the ethical concerns associated with its development and deployment. One specific concern that has been flagged in the literature is the responsibility gap (RG) due to the introduction of AI in healthcare. When the use of an AI algorithm or system results in a negative outcome for a patient(s), to whom can or should responsibility for that outcome be assigned? Although the concept of the RG was introduced in Anglo-American and European philosophy, this paper aims to broaden the debate by providing an Ubuntu-inspired perspective on the RG. Ubuntu, deeply rooted in African philosophy, calls for collective responsibility, and offers a uniquely forward-looking approach to address the alleged RG caused by AI in healthcare. An Ubuntu-inspired perspective can serve as a valuable guide and tool when addressing the alleged RG. Incorporating Ubuntu into the AI ethics discourse can contribute to a more ethical and responsible integration of AI in healthcare.

## Introduction

With the capability to perform numerous tasks, such as making autonomous decisions (Murphy, 2019), artificial intelligence (AI) is set to disrupt several industries, including the healthcare sector (Sensmeier, 2017). With AI’s potential still unfolding, it is vital to consider the ethical challenges associated with its development and deployment across the world. Yet, the attention to those ethical challenges and the perspective from which these are approached arguably do not represent an inclusive scope of global, cultural, societal and inter-relational power differentials. One specific challenge is the so-called responsibility gap (RG): if an AI system gives suboptimal, flawed or incorrect healthcare advice, can responsibility for negative outcomes be attributed to anyone? The notion of the RG was originally introduced in a philosophical debate by Matthias (2004) to indicate the concern that self-learning, autonomous systems may make it more difficult if not impossible to attribute responsibility to persons for untoward events caused by such systems. The RG has mainly—not to say exclusively—been framed through dominant philosophical perspectives. We aim to broaden the debate by providing an Ubuntu-inspired perspective on the RG debate. As Mungwini (2022) argues, other philosophical traditions can add to the pool of ideas, justifications, and stories in addition to those advanced by dominant philosophy. Indeed, as has been pointed out in feminist ethics, ‘the concept’ of responsibility may be rather misleading, in the sense that it encompasses ‘many-faceted practices’ inseparable from a context of perceptions, expectations, and judgements (Walker, 2007). A significant reason for (AI and engineering) ethics to consider an Ubuntu perspective methodologically aligns with the premise that certain sets of moral understandings cannot arbitrarily be prioritised or ignored (Walker, 2007).

Ubuntu, a concept deeply rooted in African philosophy (Mhlambi & Tiribelli, 2023; Venter, 2004), is an underrepresented perspective through which we can explore the philosophical debate on the RG. ‘The fundamental belief in *Ubuntu* is “*umuntu ngumuntu ngabantu*”’(Nzimakwe, 2014, p. 35), an isiZulu aphorism which, when translated, means ‘a person is a person through other persons’ (Metz, 2007) or more commonly, ‘I am because we are’ (Ngondo & Klyueva, 2022). This aphorism includes a descriptive component, indicating that an individual’s identity as a human being is fundamentally linked to a community. It also has prescriptive undertones, emphasising a moral requirement towards integrity, and to support the community (Metz, 2007). Ubuntu thus offers a valuable approach to address the alleged RG caused by AI in healthcare through the promotion of *collective forward-looking responsibility*. In the context of AI in healthcare, this could mean that responsibility for the consequences of AI decisions is collectively shared among a network of stakeholders (Murphy et al., 2021), which includes developers, tech companies, engineers, politicians, governments, healthcare professionals (HCPs), researchers, regulatory boards, patients and civilians. For brevity, ‘stakeholders’ will refer to any person or entity involved in the development, deployment and use of AI. Applied to the alleged RG in AI healthcare, Ubuntu stresses the importance of collective responsibility and solutions that are community-based, precisely where such responsibility might be worried to go lost due to untraceability and intractability.

This paper is structured as follows: First, we provide an overview of the current ethical debate about the alleged RG. Thereafter, we explore the notion of Ubuntu in function of the topic at hand. Third, we present an approach inspired by Ubuntu that addresses the alleged RG problem. It is important to note that this approach is inspired by Ubuntu and is not solely based on it. We must make clear that it is not our ambition to ‘theorise’ Ubuntu or present *the* systematic account of Ubuntu. It is our more moderate aim to present an ethical perspective inspired by Ubuntu as a basic collection of practical moral views that can be applied to the RG. This will enable fresh, independent, and genuine kinds of articulation (Mungwini, 2022). We conclude with an overview of practical recommendations inspired by Ubuntu, without maintaining that this provides a silver-bullet solution to the questions at hand. These modest recommendations can nonetheless inspire guidelines when addressing the alleged RG. We believe that incorporating Ubuntu into AI ethics can contribute to a more comprehensive and responsible integration of AI in healthcare.

## The Responsibility Gap: A General Idea

In conventional healthcare, HCPs (ideally) possess the required expertise to assume responsibility for the care they provide. They believe that their advice is beneficial to the patient and can explain why they think this is the case. Despite AI’s ability to calculate outputs based on inputs, AI systems in healthcare may struggle with cases outside their training data (Tsamados et al., 2021). When AI systems improve and self-learn, stakeholders have limited insight and understanding into its outcomes and potential flaws—commonly referred to as the ‘black box problem’ (Wadden, 2022)—making it difficult, if not impossible for stakeholders to assume responsibility for its actions or decisions. In AI, the black box problem refers to the fact that some AI algorithms, notably deep neural networks, lack transparency (as opposed to, e.g. linear regression models, which have been dubbed ‘white boxes’). Due to the intricacy of mathematical models and high-dimensional datasets used in deep learning (DL), it becomes impossible for humans to comprehend complex patterns and relationships (Champendal et al., 2023; von Eschenbach, 2021). Furthermore, intelligent systems that can learn from their interactions with other agents and the environment will make human prediction and control of their behaviour extremely difficult (Santoni de Sio & Mecacci, 2021). This opacity makes it hard to understand why a DL AI system comes to a particular prediction or decision, resulting in trust issues, responsibility problems and compliance with regulations.

Within AI ethics, numerous discussions on the RG often revolve around examples like self-driving cars and military robots. However, in this paper we focus on the RGs due to the introduction of AI in healthcare. For example, suppose a radiologist uses an AI system for radiology diagnosis which is accepted to be more accurate and less biased than human judgment. The radiologist suspects that a patient’s tumour is malignant, but the AI system indicates that it is benign. Trusting the AI’s superior track record, the radiologist decides against recommending a biopsy. Later, it is discovered that the tumour was indeed malignant, leading to the patient’s death due to delayed treatment. This example brings up questions about who should be responsible for misdiagnosis—either the doctor or AI developers—particularly given that the AI system itself does not have moral agency and the tasks it performs are traditionally the responsibility of humans (Lang et al., 2023; Wadden, 2022).

This brings us to the observation that concerns about the RG also seem to map on the idea that ‘responsibility as attributability’ applies only to rational entities capable of reflective self-governance pertaining to attitudes under the control of reason, which is a contentious matter as far as AI systems are concerned (Scanlon, 1998). This creates an alleged RG in terms of a disconnect or ambiguity regarding who should be held responsible for the actions, decisions, or outcomes associated with autonomous or non-autonomous AI systems. RGs may arise when there is a lack of clarity or alignment regarding the assignment of responsibility among various stakeholders involved. This is morally relevant as a lack of responsibility poses a risk of unaddressed harm. Indeed, this is where much of the appeal of utilitarian reasoning derives from: we care about how things come out, and practices of holding each other responsible are functional in securing attention on harms and benefits to others (Walker, 2007). So-called RG-pessimists are worried that this function of practices of responsibility may go lost. This is not only an ethical concern, but also a legal one, as misdiagnoses can result in additional medical costs that one of the parties involved will need to pay. While the proposed new EU directives for AI liability (the Product Liability Directive and the AI Liability Directive) are aimed at closing RGs in the context of (medical and other) AI, it has been pointed out that they only partly achieve this goal and leave several RGs intact, potentially leaving patients who are the victims of AI errors without legal recourse to pay their medical bills (Duffourc & Gerke, 2023).

AI systems’ self-learning capabilities and opacity—‘the black box problem’- have been identified as a reason for concluding that stakeholders cannot foresee consequences, and hence cannot be held responsible (Matthias, 2004; Santoni de Sio & Mecacci, 2021). Efforts to address the black box problem in AI are either aimed at avoiding it, by limiting applications to white box AI or at explaining (post hoc) how a black box algorithm came to its outputs, oftentimes referred to as* explainable AI* (XAI) (Hassija et al., 2024). Importantly, most XAI methods offer only limited transparency, as they provide hypotheses explaining why a certain input generated a certain output. These hypotheses may themselves be flawed, less precise than the original black box algorithm, and produced after the fact.

While great advancements have been made in the field of XAI, the many different approaches (attribution-based, concept-based, counterfactual-based, prototype-based, tabular-based and textual- based) all come with their own strengths and weaknesses in terms of performance and costs (for a thorough review, see Hossain et al., 2023). Therefore, white box AI appears to serve the purpose of avoiding RGs best, as it enables a HCP to ‘think along’ with the algorithm as it were (at least in as far as the company developing the white box algorithm grants the HCP access to the necessary information to interpret it, rather than resorting to intellectual property rights). However, limiting AI in healthcare to transparent models only comes at a great cost, as it would imply foregoing the implementation of AI systems based on deep neural networks, which sometimes achieve higher levels of accuracy than conventional methods in healthcare or than white box AI (Babic et al., 2021).

In fact, several authors have stated that the fixation on explainability is unnecessarily limiting the field, given that (1) It falls short of its promise to facilitate user understanding, build trust and support accountability due to the limitations mentioned above, while creating an aura of reliability and excluding promising, non-explainable AI applications (see Babic et al., 2021) and that (2) When our knowledge of causal systems is incomplete and precarious, as it often is in healthcare, the ability to explain how results are produced can be less important than the ability to produce such results and empirically verify their accuracy (see London, 2019). In line with the latter argument, Durán and Jongsma (2021) call for reliance on ‘computational reliabilism’ as an approach towards trust in medical AI while acknowledging our cognitive limitations. Yet others, e.g. Ratti and Graves (2022) emphasize the importance of XAI in healthcare applications, proposing a framework that focuses on explaining the training processes of machine learning tools. They suggest that XAI tools can, despite their shortcomings, help integrate AI technologies into broader scientific contexts, facilitating their acceptance and adoption.

For the purpose of this article, we will not adopt a strong position in the debate around XAI, but start from the observation that many stakeholders see the black box nature of certain AI applications as an obstacle for its implementation in the healthcare context, resulting in a great push towards XAI, despite its shortcomings in meeting the needs of users and communities (Meske et al., 2022). This challenge highlights the importance of ongoing collaboration to ensure that XAI aligns as much as possible with user needs and societal benefits. This is precisely where the Ubuntu approach to the RG may be useful (see below). It may be noted that the push towards transparency has moved beyond mere ‘algorithmic transparency’ towards a so-called ‘three-layer approach’ which also encompasses ‘interaction transparency’ and ‘social transparency’ (Haresamudram et al., 2023). Haresamudram et al., (2023, p. 1) refer to research that suggests that efforts to increase transparency may ‘lead to information overload, and negatively affect trust in consumers’. Their proposed alternative ‘to expand the conceptual scope of AI transparency to not only include the AI system, but also the various stakeholders interacting with the system, the context of use of the system, and the larger social implications of its continued use’ (Haresamudram et al., 2023, p. 1), may find normative backing in an Ubuntu-inspired approach.

Of note, one may wonder whether moral responsibility should presuppose that an agent possesses the ability to foresee consequences and act upon them. To be sure, practical examples of attributed responsibility suggest that this is not universally imposed across various situations. For one thing, holding parents accountable (as a proxy for responsibility) for damage incurred by their child, for instance, does not seem to match this premise. Likewise, in public and corporate contexts (including in healthcare and engineering), so-called hierarchical responsibility falls on those highest in the chain of authority even if they cannot be expected to have control over the outcomes (for an early discussion of this issue, see Thompson, 1980). According to Thompson (1980), this violates the moral presupposition that individuals should be blamed only if they could have acted otherwise. We sympathise with conceptions of moral responsibility that incorporate this presupposition but also see the ethical point in asking what goal attribution of responsibility should serve. The idea that allocation of moral responsibility rather serves a socio-moral practice of communicating what a given community disapproves, should be taken seriously. This resembles what has been called the ‘regulative function’ of practices of responsibility (Walker, 2007). In general, we side with Williams (1999) in his hesitation to adjust the various elements of responsibility into one correct conception of responsibility and leave the ambition to navigate the necessary and sufficient conditions of a coherent conception of responsibility to others.

There is, moreover, neither epistemic nor normative consensus on the question of the RG. Königs (2022, pp. 2,6), for instance, has argued that ‘[i]t is unclear whether and when responsibility gaps occur’ and that ‘if they do occur, we need not be too concerned about them’. With this, he positions himself against the ‘responsibility gap pessimists’ by stating that ‘[i]f negligent, reckless or malicious behaviour led to an autonomous system causing harm, whoever engaged in this behaviour—the manufacturer, the programmer, the operator, etc.—clearly is blameworthy’. This resonates with the position that individuals can be held responsible for the actions of AI systems.

Various authors have proposed other ways to address the RG. Some authors argue that gaining an understanding of how humans can be responsible for the actions of AI systems can be achieved through the use of collaborative agency models or principal-agent relationship models (Nyholm, 2017). Another viewpoint suggests that under specific circumstances, AI systems, similar to corporate entities, could meet the criteria to be considered responsible moral agents (List, 2021). Other authors propose tracking and tracing the actions of AI systems back to humans as a way to attribute responsibility (Santoni de Sio & Mecacci, 2021; Santoni de Sio & van den Hoven, 2018). Finally, rather than trying to ‘fill’ or ‘solve’ the responsibility gap, some believe that no RG exists (Tigard, 2021) and others that embracing RGs can be beneficial at times (Danaher, 2022).

Despite these diverse engagements with the topic and the fact that the wider field of AI ethics is slowly starting to be enriched with non-Western perspectives (Friedman, 2023; McStay, 2023; Ugar, 2022), to the best of our knowledge, no academic literature on the RG has been inspired by the notion of Ubuntu. The Institute of Electrical and Electronics Engineers (2021) stresses the importance of investigating established ethics systems from various scientific, religious, and cultural traditions. This includes considering ethical systems from Buddhism, Confucianism, African Ubuntu traditions, and Japanese Shinto influences, alongside Western philosophical traditions. The philosophy of Ubuntu can contribute to this.

## The Notion of Ubuntu

Several African philosophical discourses refer to the idea of Ubuntu (Mungwini, 2022). It is important to note that, like many fundamental ideas, Ubuntu defies a single categorisation or definition and despite being a topic of discussion for over a century, it remains a difficult—and according to some, an impossible—concept to define (Gade, 2011; Praeg, 2008). Mawere and Mubaya (2016, p. 107) have noted ‘that globally accepted practices, like democracy, are by no means universally applied but they have found their way into most communities’ and suggest that '[i]t is in the same vein that [U]buntu must find its way into every society’.

More moderately, though relatedly, we attempt to provide an overview of Ubuntu in function of the topic at hand. Ubuntu is described as a cultural and ethical framework, reflecting a way of life rather than conforming to the ‘conventional’—at least to the Western world—mould of a philosophical theory (Mawere & Mubaya, 2016). In the Zulu language of South Africa, the word Ubuntu symbolises ‘being human’. As indicated, it is based on the idea that ‘I am because we are’ (Chowdhury et al., 2021; Ngondo & Klyueva, 2022). This means that individual identities and well-being are dependent on the well-being of the community and the relationships we have with others. Nussbaum (2003, p. 21) defines Ubuntu as ‘the capacity in African culture to express compassion, reciprocity, dignity, harmony and humanity in the interests of building and maintaining community’. Ubuntu further draws attention to the acknowledgement of our responsibility to our fellow human beings and the community as a whole (Molose et al., 2018). In this sense, Ubuntu invokes an ideal of shared human subjectivity that favours community through the recognition of certain ideals, namely, community involvement and bottom-up emancipatory logic, openness, as well as harmony and partnership which—as we will discuss in subsequent sections—are relevant to both the ethical integration of AI in healthcare and its concerns, *re* the alleged RG.

This rough characterisation contains concepts that are conceptually hard to pinpoint. Notions like ‘harmony’ and ‘openness’ are difficult to descriptively delineate, at least in terms of necessary and sufficient conditions. While we sympathise with such attempts, we also realise that those concepts should, perhaps, not necessarily be approached with the one-sided ambition to describe sharp boundaries. To the extent that such notions are part of socio-moral fabrics, the onus of explanation will be on those who believe that such concepts, and the concerns that they represent, have no place in ethical deliberation. As noted by Cortina (2006, pp. 165, 166), bioethics will have to engage with ‘[t]he existence of plural moralities in everyday life’ in a process to identify ‘values and principles of a civic ethics common to the different groups’.

We also recognise that these concepts may attract normative criticism. The weight Ubuntu places on community values might draw criticism for restricting individual autonomy, while its prioritisation of openness could potentially lead to objections regarding privacy infringements or breaches of confidentiality. Likewise, Ubuntu’s promotion of harmony and partnership could be interpreted as a barrier to progress and decision-making, especially when urgent actions are required. One may counter such criticisms by arguing that Ubuntu’s focus on community values does not restrict individuality but rather encourages recognising individual autonomy within the community. While its focus on openness could potentially lead to objections regarding privacy infringements or breaches of confidentiality, Ubuntu promotes open dialogue and respectful disagreement while seeking consensus for collective agreement. Furthermore, although Ubuntu’s promotion of harmony and partnership may lead to slow decision-making in situations of urgency, Ubuntu upholds a balance between inclusivity and efficiency. It may be suggested that at least as ideals, these notions can counterweigh narratives that foster a standard to ‘move fast and break things’.

## Ubuntu and its Role in Addressing the RG

It is worthwhile to note the alleged RG encompasses several elements such as the backwards-looking element of determining responsibility after a negative outcome occurs, the opacity of AI systems making it difficult to explain their decisions, and the tendency to attribute responsibility to individual stakeholders. Ubuntu offers valuable insights to address these elements by promoting community involvement and bottom-up emancipatory logic in AI development and deployment, encouraging transparency and openness in algorithm design and honesty about its limitations to enhance collective forward-looking responsibility, as well as promoting partnerships and harmony to balance interests and values. This fosters a sense of shared responsibility that goes beyond individual blame.

Therefore, addressing the RG from an Ubuntu perspective may emphasise the notion of actively taking up responsibility (as opposed to being attributed responsibility and potentially blame by others), as it seems to place particular emphasis on ‘due care’^1^ for the community. Put simply, Ubuntu highlights a duty of care towards the community in which one is active. In Ubuntu, this would mean protecting the welfare of the community and assuming responsibility when that protection has been comprised. This can also help to bridge the gap between personal responsibility and social justice (Mayaka & Truell, 2021), by encouraging individuals to understand their actions in the context of the wider community and to take steps to address social issues in a collaborative, forward-looking manner.

This emphasis that Ubuntu places on due care is particularly relevant in the discussion of alleged RGs, as responsibility is not merely ‘backwards-looking’—say, a ‘whodunit’ looking for the nearest human factor that can be blamed. Responsibility is also—and perhaps especially—about due care, i.e., behaving ‘responsibly’. In healthcare, professionals are not only expected to retrospectively account for harm caused by mistakes (backwards-looking), but also—and more importantly—to apply due diligence and take proactive actions to explain diagnoses and prevent (future recurrences of) errors (forward-looking). Thus, rather than observing that RGs may render it difficult to *appoint* responsibility to any particular stakeholder(s), it may be more fruitful to consider broader notions of interpersonal and global responsibilities, focussing on the actions and efforts required to bring about a desirable state of affairs (forward-looking), rather than providing a causal explanation of the responsible agent (backwards-looking). Collective forward-looking responsibility, similar to its backwards-looking equivalent, refers to the responsibility of a collective agent for a specific state of affairs in the world. However, unlike the backwards-looking variant, it ‘does not focus on whether a particular collective agent caused harm in the sense relevant to moral blameworthiness’ (Smiley, 2023, para. 4).

Collective forward-looking responsibility is not without criticism as ‘it is still unclear whether collectives can become (moral) agents and how collective action and intention are possible at all’ (Müller et al., 2021, p. 4). Some scholars argue that responsibility can only be constructed in individual terms, and that ‘collective responsibility’ is merely exaggerated individual responsibility (Smiley, 2023). Likewise, the idea that responsibility can be essentially shared—like in Young’s forward-looking social connection model of responsibility—seems to imply that everyone who sustains a certain societal practice can be held responsible. This can make a very heavy burden and it is unclear to us whether Ubuntu can escape this concern. While this is something to consider, it should also be noted that the reason why this theoretical issue ensues, is tied to the very reason that makes it worthwhile to consider alleged RGs through an Ubuntu lens in the first place. Through such a perspective, in which distribution of responsibility is generalised, mapping the ‘geography of responsibility’ (Walker, 2007) would have us asking: ‘Why did this situation (an alleged RG) occur in the first place?’, ‘What can be done to ensure that similar situations do not occur in the future?’, ‘How can we, presently, care for the victim/s of this situation?’, ‘Who do we rely on to ensure that AI systems in healthcare are designed for the benefit of the community?’ and ‘How do we ensure that AI systems in healthcare yield positive results?’.

Providing a comprehensive account of (dis)similarities between Ubuntu and Western moral theories, as well as other renditions of collective responsibility, would be a long and difficult task because such an account will face the paralysing heterogeneity of attempts to display Ubuntu and the variations that exist within other moral theories. We cannot attempt this here, but we can broach some interesting observations that we think stand out for further analysis. Discussions of similarities between Ubuntu and Western moral theories (virtue ethics, Kantian deontology, and utilitarianism) can be found in Mawere and Mubaya (2016), and notions of how it bears similarities with ethical theories located within ethics of care can be found in Hall et al. (2013).

## Applying Ubuntu to the RG

We have already noted that some aspects of an Ubuntu-inspired approach to the RG may align with Young’s (2011) social connection model of responsibility, implying a shared obligation to transform the structural processes that lead to certain adverse events. More generally, care ethics seeks to maintain relationships by contextualising and promoting the well-being of community members in a network of social relations (Sander-Staudt, 2011), which is similar to Ubuntu. Robert Goodin’s (1985) ethics of responsibility may also be noted as a normative view with circumspect attention to people's responsibilities distinct from ‘traditional’ deontological, utilitarian and aretaic approaches, which sets out to define extensive individual and collective obligations to ‘unknown and unknowable strangers’ (Goodin, 1985; Walker, 2007). It has been argued that this account may ultimately fail to ground these obligations, mainly because it is unclear whether the required sorts of ‘responsibility-entailing connections’ actually exist (Walker, 2007). In our view, an Ubuntu-inspired approach may circumvent this, since it is normatively premised upon a communal ideal that prioritises the well-being of all individuals while fostering a sense of *collective* and *forward-looking* responsibility. In exploring ways to address RGs in AI, the importance of taking an Ubuntu-inspired approach seriously will minimally lie in how it can foreground certain facts of dependency and connection, which dominant philosophical views of responsibility tend to eclipse (Walker, 2007).

Embracing an Ubuntu-inspired approach, we can navigate the ethical challenges associated with AI in healthcare, *re* RG. Collective forward-looking responsibility, within the framework of Ubuntu, involves recognising that all stakeholders share the responsibility for shaping the future of AI in healthcare. It requires a collaborative effort which can lead to a greater understanding of how individual actions can have a ripple effect on the wider community, and to a sense of obligation to work *collectively* and *forwardly* to address the alleged RG. Here are a few ways this can be done:

i. Community involvement and bottom-up emancipatory logic—The Ubuntu ideal of community involvement and bottom-up emancipatory logic highlight the interconnectedness of individuals within a community. This fosters collective well-being through active collaboration, and shared responsibility in decision-making (Chigangaidze et al., 2022; Ujomudike, 2016). Encouraging the participation and input of a diverse range of stakeholders, including subject matter experts, community members, and representatives from marginalised groups, can help ensure that the impact of AI systems is thoroughly considered and that the perspectives of all affected stakeholders are taken into account. This could serve as an alternative to the traditional top-down, hierarchical approach to designing and implementing AI systems. For example, community involvement may put a face to the consequences of RGs in practice and give a voice to the victims of AI system errors for which nobody takes responsibility, which may in turn lead to initiatives to prevent such gaps where possible, or alternatively provide help for victims from a collective responsibility perspective.
ii. Openness—The ideal of openness promotes the distribution of knowledge to foster mutual cooperation and trust. Ensuring that AI systems are transparent and that the decision-making processes they employ can be traced and audited can help ensure accountability and promote trust in these technologies. To the extent that such transparency cannot (sufficiently) be obtained—due to black box or grey box concerns—an Ubuntu-inspired perspective can prod the duty to preserve and cultivate the competence to evaluate why a system reaches a given outcome and to assess value alignment in the light of respecting patients’ autonomy. For example, a situation in which patients know the limitations of their HCP’s understanding of the AI algorithm used in their care, understand the pros and cons of using it and decide that they prefer its use, is preferable over vague and potentially misleading concepts promising ‘trustworthy AI’ and discounting its limitations.
iii. Harmony and Partnership—The ideals of harmony and partnership encourage peaceful cooperation, while also seeking to resolve conflicts through discussion, understanding, and reconciliation. Fostering collaboration and partnerships between stakeholders can help ensure that the development and deployment of AI systems are guided by a shared sense of responsibility and a commitment to the common good. For example, in case medical errors are the consequence of an AI tool being employed by a well-meaning HCP, looking for solutions and support for the victim, rather than focussing on blame can lead to a better outcome for all parties involved.

These ideals may help address the RG. One way that this can be done is by incorporating this Ubuntu-inspired approach into ethical guidelines. This might inspire societies that embrace AI systems in healthcare to be designed and used in ways that are responsible, fair, and respectful to all members of society. The utility of ‘guidelines’ is not without criticism, though through the lens of Ubuntu, more emphasis may be put on the observation that such guidelines are ‘the work of human beings’ and that such rules are open to revision and interpretation, as new information comes in (Davis, 1999). Also, acting responsibly would not be limited to methodically ticking off boxes from a list of guidelines, but would include acting with genuine concern, as Ubuntu would have it, for everyone affected by a new AI technology.

## Conclusion

As we embrace the transformative potential of AI in healthcare, it is essential to acknowledge and address the ethical concerns associated with its implementation. The RG arising from negative outcomes caused by AI systems in healthcare necessitates careful consideration of responsibility. The Ubuntu-inspired approach offers a collective forward-looking approach to tackle the alleged RG in AI healthcare. Furthermore, it enriches the AI ethical discourse by infusing the moral ideals of community involvement, bottom-up emancipatory logic, openness, and harmony into the decision-making processes related to AI in healthcare. We hope that this Ubuntu-inspired approach will encourage future philosophical and empirical work on alleged RGs from understudied representations, including Ubuntu. Now more than ever it becomes expedient to have a wider representation of ethical perspectives that accounts for diverse ethos and contexts.

## Footnotes

1 Due care, in this case, refers to the level of responsibility expected from an individual within the community.
